# Increased ATP generation in the host cell is required for efficient vaccinia virus production

**DOI:** 10.1186/1423-0127-16-80

**Published:** 2009-09-02

**Authors:** Chia-Wei Chang, Hui-Chun Li, Che-Fang Hsu, Chiao-Yen Chang, Shih-Yen Lo

**Affiliations:** 1 Graduate Institute of Molecular and Cellular Biology, Tzu Chi University, Hualien, Taiwan, Republic of China; 2 Graduate Institute of Medical Sciences, Tzu Chi University, Hualien, Taiwan, Republic of China; 3 Graduate Institute of Medical Biotechnology, Tzu Chi University, Hualien, Taiwan, Republic of China; 4 Department of Laboratory Medicine, Buddhist Tzu Chi General Hospital, Hualien, Taiwan, Republic of China

## Abstract

To search for cellular genes up-regulated by vaccinia virus (VV) infection, differential display-reverse transcription-polymerase chain reaction (ddRT-PCR) assays were used to examine the expression of mRNAs from mock-infected and VV-infected HeLa cells. Two mitochondrial genes for proteins that are part of the electron transport chain that generates ATP, *ND4 *and *CO II*, were up-regulated after VV infection. Up-regulation of ND4 level by VV infection was confirmed by Western blotting analysis. Up-regulation of ND4 was reduced by the MAPK inhibitor, apigenin, which has been demonstrated elsewhere to inhibit VV replication. The induction of ND4 expression occurred after viral DNA replication since ara C, an inhibitor of poxviral DNA replication, could block this induction. ATP production was increased in the host cells after VV infection. Moreover, 4.5 μM oligomycin, an inhibitor of ATP production, reduced the ATP level 13 hr after virus infection to that of mock-infected cells and inhibited viral protein expression and virus production, suggesting that increased ATP production is required for efficient VV production. Our results further suggest that induction of ND4 expression is through a Bcl-2 independent pathway.

## Background

Vaccinia virus (VV), a member of the *Poxviridae *family, is an enveloped, DNA virus with a genome of 192 kb encoding about 200 proteins [1]. Various cell lines can be infected by VV, including HeLa, CV-1, mouse L, and chicken CEF cells [2,3]. VV causes major changes in host cell machinery shortly after infection, and cytopathic effects (CPE) are observed several hours after infection with VV [2-4]. VV infection modulates host cell gene expression: several previous studies have shown that mRNA synthesis in the host cells was inhibited immediately after VV infection [5,6]. Microarray analysis showed that around 90% of the host genes were down-regulated after VV infection, including genes involved in DNA replication, transcription, translation, apoptosis, and the proteasome-ubiquitin degradation pathway [7,8]. Only a smaller fraction of host genes were up-regulated after VV infection, including WASP protein, and genes implicated in immune responses [7,8].

Several viral factors of VV utilize ATP and several steps in viral multiplication of VV require ATP [9-14]. ATP is also required for DNA packaging and capsid maturation of herpes simplex virus, for capsid assembly and release of type D retrovirus, for capsid assembly of human immunodeficiency virus, and for budding of influenza virus [15-18]. Therefore, it was expected that viral factors would modulate cellular energetics to benefit the virus, though this area is understudied [19].

In this study, the possible up-regulation of host cell genes after VV infection was analyzed by differential display-reverse transcriptase-polymerase chain reaction (ddRT-PCR), a simple technique with high sensitivity and specificity . Two mitochondrial genes involved in the electron transport chain (*ND4 *and *COII*) to generate ATP were found to be up-regulated after VV infection using this assay.

## Materials and methods

### Cell culture

HeLa cells, MDCK cells, HuH7 cells and Con1 cells with full-length HCV genome were cultured in Dulbecco's modified Eagle's medium (DMEM) containing 10% fetal bovine serum (FBS),100 U/ml penicillin and 100 μg/ml streptomycin (Gibco, USA) [20]. HCV sub-genomic replicon cells were cultured in DMEM with 10% FBS, 100 U/ml penicillin, 100 μg/ml streptomycin, and 400 μg/ml G418 [21]. HepG2 and 1.3 × ES2 HepG2 (HBV) were cultured in DMEM containing 10% FBS, 100 U/ml penicillin,100 μg/ml streptomycin and 1% non-essential amino acids (Gibco, USA) [22]. All cultured cells were maintained at 37°C with 5% CO_2_.

### Virus infection

Vaccinia virus WR strain was used to infect HeLa cells in this study, following previously published procedures for virus amplification and plaque assay [23,24]. Cytosine arabosinide (ara C), where used, was added to the cells at a concentration of 40 μg/ml [25].

Influenza A virus WSN33 was used to infect MDCK cells following previously published procedures for virus amplification and plaque assay [26].

### Plasmid construction and DNA transfection

To clone the DNA fragment for *N1L *gene coding region, vaccinia genomic DNA was used as template and forward and reverse PCR primers (5'-CGGAATTCATGAGGACTCTACTTAT-3' and 5'-TGCTCTAGATTTTTCACCATATAGATC-3') were used to amplify the gene fragment. After PCR, the DNA fragment was digested by restriction enzymes (*Eco*RI/*Xba*I) and cloned into the expression vector pcDNA3.1-V5-His A (linearized by *Eco*RI/*Xba*I). This expression plasmid was verified by sequencing. An Exgen 500 kit (Fermentas, USA) was used to transfect DNA into HeLa cells following the manufacturer's instructions.

### RNA extraction and ddRT-PCR

Total RNAs were extracted from HeLa cells 21 hr after VV infection (MOI = 1) using an RNeasy Mini kit (Qiagen, Germany) following the manufacturer's instructions. The ddRT-PCR assay was performed using a GeneFishing DEG Premix kit (Seegene, Korea), following the manufacturer's instructions.

### Western blotting analysis

Our previous procedures were followed for Western blotting analysis [27,28]. A rabbit polyclonal antibody against ERK-2, a mouse monoclonal antibody against Bcl-2 and goat antibodies against ND4 and COII were purchased from Santa Cruz Biotechnology (USA). Antibodies against SDHA, ATP5O, SDHB, COVc were purchased from Abcam company (UK). Rabbit antibodies against vaccinia viral proteins (A type inclusion protein and IMV heparin binding surface protein) were generated in the lab.

### Measurement of ATP production

Ten hours after 2 × 10^5 ^HeLa cells were seeded in one 35-mm culture dish, cells were infected with VV (MOI = 1 or 5). Intracellular ATP was then analyzed at different time points (virus-infected cells versus non-infected cells) using the ATP bioluminescence assay kit HS II (Roche, Germany) to determine ATP.

### RNAi experiments

RNAi experiments were performed using the lentiviral expressing system , following the manufacturer's instructions. RNAi reagents were obtained from the National RNAi Core Facility located at the Institute of Molecular Biology/Genomic Research Center, Academia Sinica.

## Results

### Elevated expression of mitochondrial genes *ND4 *and *COII *after vaccinia virus infection

**Figure 1:**
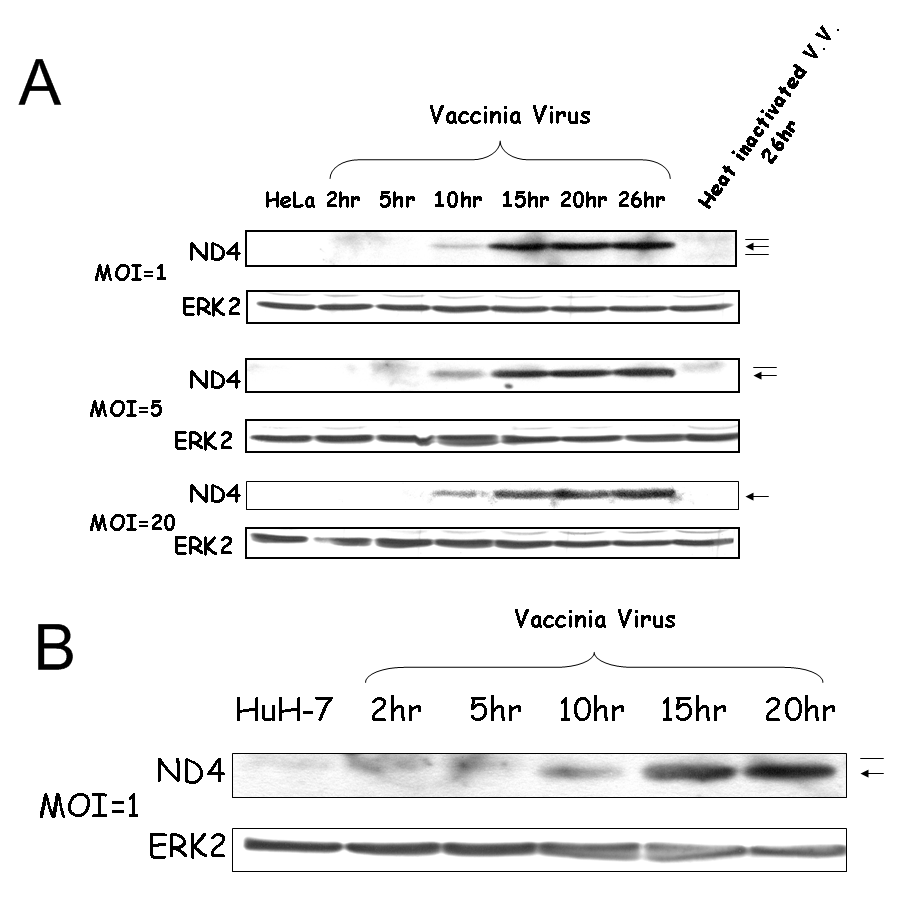
**Western blotting analysis of ND4 expression in vaccinia virus infected cells**. (A) The HeLa cells were infected with MOI = 1 (or 5 or 20) of vaccinia virus for various time periods, as indicated. ND4 expression (marked by an arrow) was analyzed by Western blotting. ERK2 protein was served as a loading control. Occasionally, two non-specific bands (one larger and one smaller than ND4, indicated by thin lines) were detected in the samples. (B) ND4 was also up-regulated in HuH7 cells after vaccinia virus infection. The ND4 protein is marked by an arrow while a non-specific band is indicated by a thin line.

To search for cellular genes up-regulated after vaccinia virus infection, ddRT-PCR assay was performed to examine the expression levels of mRNAs from mock-infected and infected HeLa cells (Additional file 1). Four DNA fragments differentially expressed after virus infection were cloned: two fragments were VV genes while the other two were the mitochondrial genes *ND4 *and *COII*. Induction of ND4 expression in HeLa cells by VV infection was also confirmed by Western blotting (Fig. 1A). ND4 protein expression in HeLa cells increased 10 hr after vaccinia virus infection. Furthermore, up-regulation of ND4 expression by VV infection was also observed in HuH7 cells (Fig. 1B). Thus, the expression of the mitochondrial gene *ND4 *was up-regulated after VV infection in different host cells. Similar up-regulation of host ND4 expression was not detected when VV was heat-inactivated before infection (Fig. 1A), after influenza A virus infection, or in the presence of hepatitis B viral genome or hepatitis C viral RNA (Additional file 2).

### Apigenin and AraC blocked ND4 induction after vaccinia virus infection

**Figure 2:**
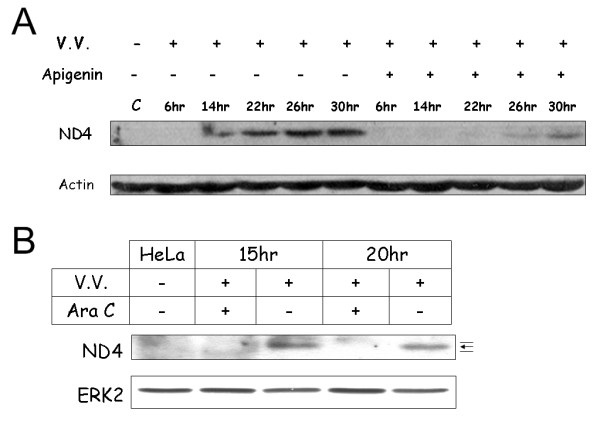
**Western blotting analysis of drug effects on ND4 expression in VV infected cells**. (A) HeLa cells were infected with VV (MOI = 1) for the indicated times in the presence or absence of 45 μM apigenin. Actin was used as a loading control. (B) The ND4 up-regulation by vaccinia virus infection (MOI = 1) was inhibited by the DNA polymerase inhibitor, ara C. Cells were incubated with ara C (40 μg/ml), an inhibitor of poxviral DNA synthesis, 30 min before VV infection and throughout the infection. The ND4 protein is indicated with an arrow while non-specific bands are indicated by thin lines. ERK2 protein was used as a loading control.

Previous studies have shown that the VV-stimulated mitogen-activated protein kinase (MAPK) pathway is required for virus multiplication [25,29]. The MAPK pathway inhibitor apigenin was used to analyze the effect of MAPK blockade on the up-regulation of ND4 expression following VV infection (Fig. 2A) [30]. Apigenin (45 uM) significantly reduced the amount of ND4 expression up-regulation induced by VV infection. The effects of the nucleotide analogue Ara C, which blocks the replication of vaccinia virus, on the up-regulation of ND4 expression were also examined [25]. As shown in Fig. 2B, ara C (40 μg/ml) blocked the increase in ND4 expression induced by VV infection.

### Analysis of several nuclear genes encoding mitochondrial electron transport chain proteins after VV infection

**Figure 3:**
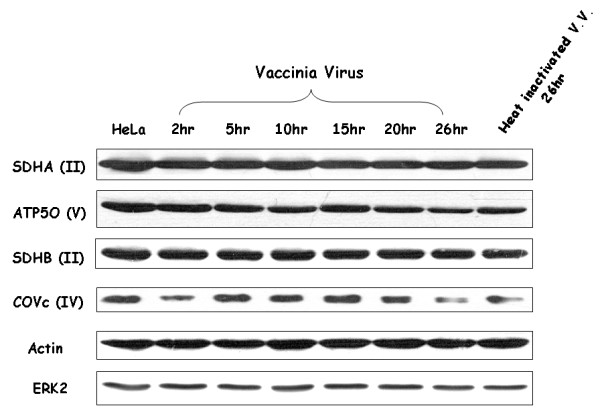
**Analysis of several nuclear genes encoding mitochondrial electron transport chain proteins after VV infection by Western blotting analysis**. The Roman numbers in parentheses represent the respiratory complex in which the gene participates. ERK2 protein was used as a loading control.

Both *ND4 *and *COII *are mitochondrial genes whose protein products are part of the electron transport chain that generates ATP in cells [31]. To determine whether other mitochondrial electron transport chain proteins encoded in the nucleus are also up-regulated after VV infection, protein expression of four randomly selected genes involved in electron transport chain was analyzed using Western blotting analysis. Compared to the expression of β-actin gene or ERK2 gene, no significant differences were observed after VV infection (Fig. 3).

### Intracellular ATP generation increased after vaccinia virus infection

**Figure 4:**
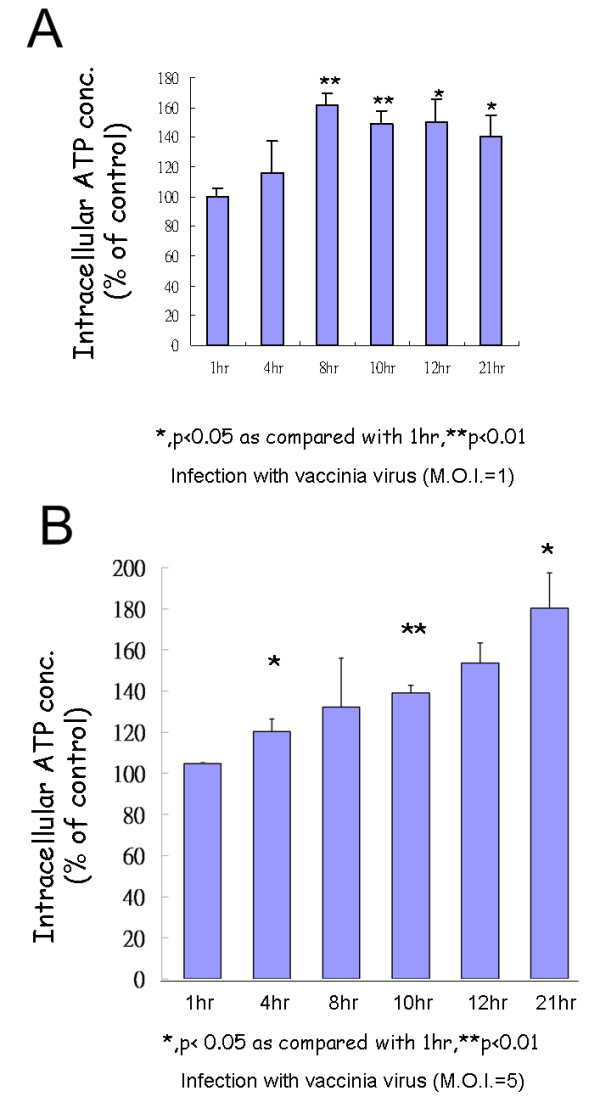
**The intracellular ATP concentration was significantly increased after VV infection**. Intracellular ATP concentration was measured at the indicated times (1, 4, 8, 10, 12, and 21 hr) after vaccinia virus infection in HeLa cells (MOI = 1 in Fig. 4A and MOI = 5 in Fig. 4B). Experiments were performed three times in duplicate.

As the above results (Fig. 1 and Fig. 3) suggest that at lease some of the proteins involved in the electron transport chain to generate ATP are up-regulated after VV infection, the intracellular ATP concentration was measured. Compared to mock-infected cells, ATP production was significantly higher in host cells after virus infection (Fig. 4A and Fig. 4B). Using M.O.I. = 1 of vaccinia virus (Fig. 4A) for infection, compared to the ATP level of mock-infected HeLa cells at each time point (as 100%), the ATP level 1 hr after virus infection was 99.7%, 116% after 4 hr (*P *= 0.324), 161% after 8 hr (*P *= 0.001), 149% after 10 hr (*P *= 0.002), 150% after 12 hr (*P *= 0.011), 141% after 21 hr (*P *= 0.018). Using M.O.I. = 5 of vaccinia virus (Fig. 4B) for infection, compared to the ATP level of mock-infected HeLa cells at each time point (as 100%), the ATP level 1 hr after virus infection was 104.6%, 120.3% after 4 hr (*P *= 0.035), 132.1% after 8 hr (*P *= 0.193), 139.2% after 10 hr (*P *= 0.003), 153.7% after 12 hr (*P *= 0.078), 180.3% after 21 hr (*P *= 0.018).

### Oligomycin, an inhibitor of ATP generation, reduced the production of vaccinia virus

**Figure 5:**
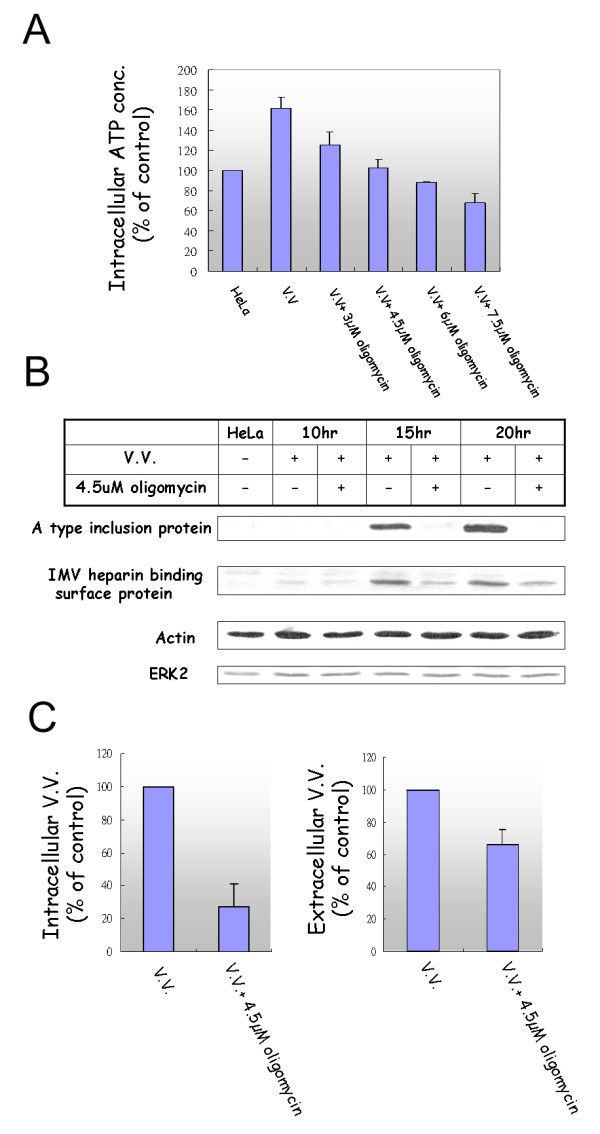
**(A) The intracellular ATP concentration of vaccinia virus infected (MOI = 1) and oligomycin-treated cells**. ATP concentration was measured 13 hrs after vaccinia virus infection in the presence of various concentration of oligomycin. Experiments were performed three times in duplicate. (B) Western blotting analysis of Vaccinia viral protein expression (A type inclusion protein and IMV heparin binding surface protein) in the presence of 4.5 μM oligomycin. ERK2 protein was used as a loading control. (C) Both the intracellular and the extracellular vaccinia viruses were reduced 13 hr after virus infection in the presence of 4.5 μM oligomycin. Experiments of plague assay were performed three times in triplicate.

To determine whether this increased ATP production after VV infection is necessary for virus production, oligomycin, an inhibitor of ATP production, was used [32]. Compared with the ATP level of non-treated HeLa cells (as 100%), the ATP level of virus-infected cells (M.O.I. = 1) was 162% without oligomycin, 125% with 3 μM oligomycin, 103% with 4.5 μM oligomycin, 88% with 6 μM oligomycin, 68% with 7.5 μM oligomycin. Thus, treatment with oligomycin at 4.5 μM reduced the ATP level in virus-infected cells to that of mock-infected cells 13 hr after infection (Fig. 5A). Therefore, 4.5 μM oligomycin was used to evaluate the effect of increased ATP generation on viral protein expression and virus production. Indeed, the expression of two viral proteins (A type inclusion protein and IMV heparin binding surface protein) was suppressed in the presence of 4.5 μM oligomycin (Fig. 5B). The number of intracellular mature virions was reduced to about 28% (*P *= 0.01162) and extracellular enveloped virions to about 66% of control (*P *= 0.022722) (Fig. 5C) 13 hrs after virus infection. In contrast, the replication of HCV sub-genomic RNA was not affected by 3, 6, 9, or 12 μM oligomycin (Additional file 3). Typically, oligomycin has been used to block ATP production at a concentration greater than 20 μM [32]. The intracellular ATP concentration of HeLa cells treated with 25 μM oligomycin was reduced to about 50% that of non-treated cells (Additional file 4). When VV-infected cells were treated with 20 to 35 μM oligomycin, the intracellular ATP concentration was reduced to about 50% of mock-infected cells 13 hr after virus infection (Additional file 4), and the intracellular mature virions were reduced to about 10% of the level in non-treated cells (data not shown).

### Vaccinia virus infection induces ND4 expression through a Bcl-2 independent pathway

**Figure 6:**
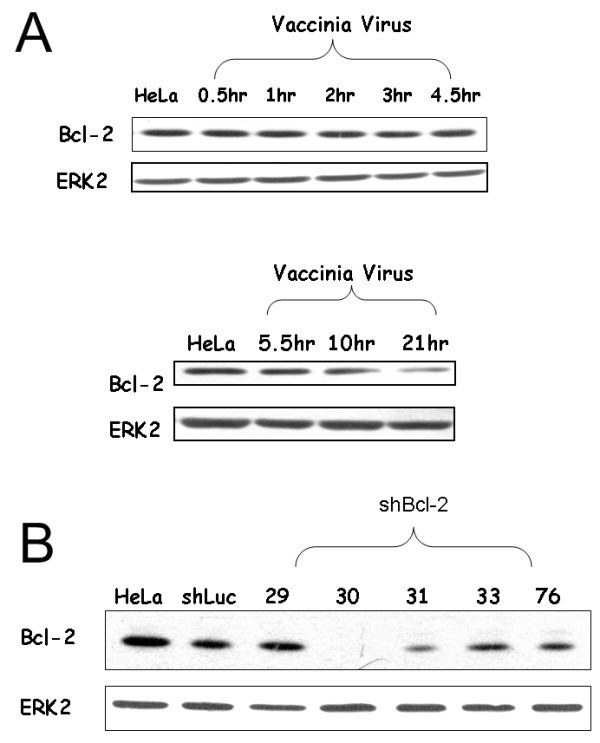
**(A) The expression of Bcl-2 was not elevated during vaccinia virus infection**. HeLa cells were infected with VV (MOI = 1) for the indicated times, then cell lysates were analyzed by Western blotting. ERK2 protein was used as a loading control. (B) Western blotting analysis of Bcl-2 in HeLa cells stably transfected with different shRNA clones against Bcl-2. HeLa cells with shRNA against the Luciferase gene were used as a control. ERK2 protein was used as a loading control.

**Figure 7:**
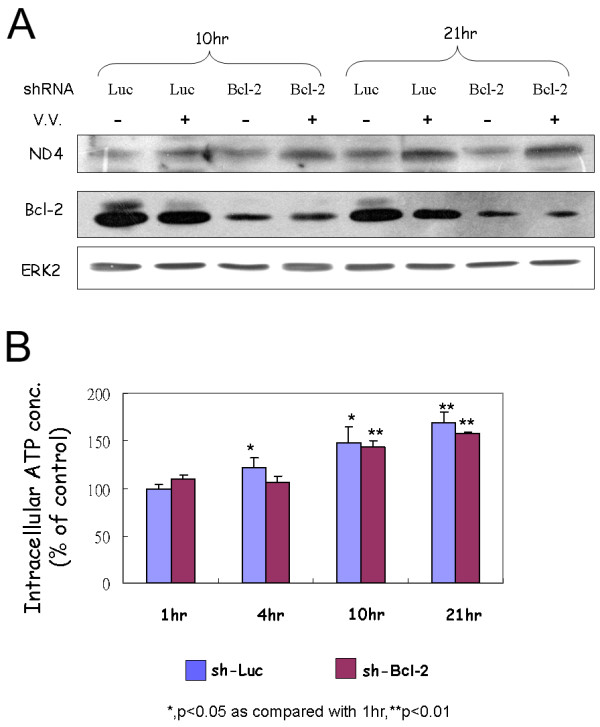
**(A) Western blotting analysis of ND4 protein in vaccinia virus-infected HeLa cells with shRNAs against Luc or Bcl-2**. ERK2 protein was used as a loading control. (B) Intracellular ATP concentration was measured at the indicated times (1, 4, 10, and 21 hr) after vaccinia infection of HeLa cells (MOI = 1). Experiments were performed three times in duplicate.

The Bcl-2 family of proteins are implicated in regulating cellular bioenergetics, perhaps by regulating the availability of mitochondrially produced ATP [33,34]. Western blotting analysis was used to examine whether Bcl-2 expression is involved in the elevated ATP production after VV infection. There was no up-regulation of Bcl-2 in a comparison of mock-infected and virus-infected cells at different time points. Moreover, Bcl-2 expression clearly decreases at 21 hr post-infection when ATP production and ND4 expression is optimally increased (Fig. 6A). Different shRNA clones against Bcl-2 were used to knock-down its expression in HeLa cells (Fig. 6B). Clone 30 of these shRNAs almost knocked-down Bcl-2 expression entirely. However, HeLa cells stably transfected with this shRNA could not survive anymore (data not shown). Thus, HeLa cells with shRNA clone 31 against Bcl-2 were used to evaluate the effect of Bcl-2 on the up-regulation of ND4 and ATP generation after VV infection. Expression of Bcl-2 protein in these cells was not affected by VV infection at different time points (Additional file 5). In these Bcl-2 knock-downed cells, up-regulation of ND4 after VV infection was still observed (Fig. 7A). ATP generation was also increased after VV infection in these cells (Fig. 7B).

Vaccinia virus N1L protein was reported to have a similar secondary (though not primary) structure to Bcl-2 protein [35]. To determine whether N1L may be responsible for the up-regulation of ND4 and COII after VV infection, the *N1L *gene fragment was cloned and expressed in the cells. No up-regulation of ND4 or COII was detected in the presence of N1L protein (Additional file 6).

## Discussion

In this study, at least some of the genes involved in ATP generation were found to be up-regulated after VV infection (Fig. 1). Only two viral genes were detected in our ddRT-PCR assay among about 200 proteins vaccinia virus encoded (Additional file 1). Thus, only a small portion of differentially genes was identified by this assay. Therefore, it is not surprising that only *ND4 *and *COII *genes rather than all 12 mRNA molecules in the same polycistronic transcript were identified [31]. Host cell genes up-regulated after vaccinia virus infection found in this study were not the same as those found in previous microarray assays, which suggests that ddRT-PCR and microarray assays should be used in tandem for a more complete analysis of differential gene expression between mock-infected and virus-infected cells [7,8].

Our results demonstrate that ATP generation did increase after vaccinia virus infection (Fig. 4). Our results (Figs. 1, 3, and 4) also indicate that increased ATP generation did not require the up-regulation of all the proteins involved in mitochondrial electron transport chain. Up-regulation of mitochondrial-encoded rather than nuclear-encoded proteins involved in mitochondrial electron transport chain could increase ATP generation. This may suggest that mitochondrial-encoded proteins rather than nuclear-encoded ones involved in electron transport chain are rate-determining proteins in ATP generation.

Our results demonstrate that increased ATP production is essential for efficient VV production (Fig. 5). It is not surprising that viral factors would modulate cellular energetics to benefit the virus, though this area is understudied [19]. To our knowledge, this study is the first report to demonstrate that virus infection could up-regulate the expression of genes involved in ATP generation and increase ATP production. It is interesting to note that the intracellular mature virions were more sensitive than the extracellular enveloped virions to oligomycin treatment (Fig. 5C). This may suggest that the assembly of intracellular mature virions is more ATP-dependent [1].

ATP is required for the budding of influenza virus [16]. Similar up-regulation of ND4 expression after VV infection was not detected after replication of hepatitis B and C viruses or infection with influenza A virus (Additional file 2). One possibility is that there was no need to increase ATP generation because there was already enough existing energy source for multiplication of these viruses. Alternatively, increased ATP generation in cells may still occur here after infection with these viruses though the up-regulation of ND4 is smaller than that caused by VV infection.

No amplification of mitochondrial DNA after vaccinia virus infection was observed (data not shown). Therefore, the up-regulation of ND4 after virus infection is possibly through transcriptional regulation. However, little is known about the regulation of mitochondrial transcription [31]. Bcl-2 family proteins are implicated in regulating cellular bioenergetics, perhaps by regulating the availability of mitochondrially produced ATP [33]. However, the amount of Bcl-2 protein in the host cell did not increase after VV infection (Fig. 6A). Induction of ND4 protein and increased ATP generation are still occurred after VV infection in HeLa cells with reduced amount of Bcl-2 protein (Fig. 7A and 7B). VV N1L protein has been reported to have similar secondary structure as Bcl-2 protein, but not primary structure, and may influence ATP levels in vivo [35,36]. However, neither host *ND4 *nor *COII *genes were up-regulated by N1L protein (Additional file 6), which is consistent with a previous report showed that N1L did not interact with mitochondria [37]. These results indicate that increased ATP generation after VV infection is a Bcl-2 independent event. Further studies are needed to clarify the mechanism(s) of the up-regulation of genes involved in ATP production after VV infection.

The up-regulation of ND4 expression was reduced by apigenin and ara C (Fig. 2), suggesting that event(s) occurring after viral DNA replication are responsible for the up-regulation of ND4 expression. These results are in agreement with the observation that neither *ND4 *nor *COII *gene was up-regulated by N1L protein (Additional file 6) since N1L is an early gene product [1].

## Conclusion

In summary, at least some of genes involved in ATP generation were up-regulated by VV infection, and in turn, cellular ATP generation was increased. This increased ATP generation was required for efficient VV production.

## Competing interests

The authors declare that they have no competing interests.

## Authors' contributions

CWC conducted the experiments and analyzed the data, HCL analyzed the data and wrote the manuscript, CFH conducted the experiment of Fig. 4B, CYC made the polyconal antibodies against vaccinia virus, and SYL designed the experiments and wrote the manuscript. All authors read and approved the final manuscript.

## Supplementary Material

Additional file 1 **Supplementary Fig. S1 - Gel analysis of ddRT-PCR products**. DNA samples in different lanes represent the outcomes of different sets of primers for PCR (only four sets of primers out of 20 are shown). DNA fragments (marked by arrows) selected to perform T/A cloning and sequencing were cytochrome c oxidase subunt II gene product (lane 2); VV gene products (lanes 4, 6); NADH dehydrogenase subunit IV gene product (lane 8). C: mock-infected HeLa cell; V: 21 hr after vaccinia virus infection (MOI = 1) in HeLa cells. Click here for file

Additional file 2 **Supplementary Fig. S2. - Western blotting analysis of ND4 protein expression**. Samples were prepared from VV-infected HeLa cells, influenza A virus-infected MDCK cells, HCV replicon cells (with sub-genomic HCV RNA), con1 cells (with full-length HCV RNA) and 1.3 × ES2 cells (with HBV genome). Mock-infected (MDCK cells) or non-transfected parental cells (HuH7 and HepG2 cells) were used as controls. ERK2 protein was used as a loading control. Click here for file

Additional file 3 **Supplementary Fig. 3. - Western blotting analysis of NS5A to reflect the amount of HCV sub-genomic RNA**. Lane 1, parental HuH7 cell; lane 2, HCV sub-genomic replicon without oligomycin; lane 3, HCV sub-genomic replicon with 3 μM oligomycin; lane 4, HCV sub-genomic replicon with 6 μM oligomycin; lane 5, HCV sub-genomic replicon with 9 μM oligomycin; lane 6, HCV sub-genomic replicon with 12 μM oligomycin. Western blotting was performed 24 hr after oligomycin treatment. Erk-2 protein was used as a loading control. Click here for file

Additional file 4 **Supplementary Fig. 4. - Intracellular ATP concentration was measured 13 hr after VV infection (MOI = 1) in the presence of different oligomycin concentrations**. Experiments were performed in duplicate. Compared with the ATP level of non-infected and non-treated HeLa cells (as 100%), the ATP level of virus-infected cells was 164% without oligomycin, 49% with 20 μM oligomycin, 52% with 25 μM oligomycin, 49.5% with 30 μM oligomycin, and 52.6% with 35 μM oligomycin. The ATP level was reduced to 58.5% in the presence of 25 μM oligomycin in mock-infected HeLa cells. Click here for file

Additional file 5 **Supplementary Fig. 5. - Western blotting analysis of Bcl-2 expression in vaccinia virus infected cells**. The HeLa cells stably transfected with either shLuc or shBcl-2 (clone 31) were infected with MOI = 1 of vaccinia virus for various time periods, as indicated. ERK2 protein was served as a loading control. Click here for file

Additional file 6 **Supplementary Fig. 6. - Western blotting analysis of ND4 and COII expression in N1L expressed cells**. HeLa cells were transfected with expression vector only (pcDNA3.1-V5-HisA) or with N1L expression plasmid (N1L protein with a V5 tag). Cell lysates were analyzed by Western blot 48 hr after transfection. The cell lysate from HeLa cells 28 hr after vaccinia virus infection was used as a positive control, and ERK2 protein was used as a loading control. Click here for file
