# Supplementary figures and images for: Increased ATP generation in the host cell is required for efficient vaccinia virus production
Source: J Biomed Sci. 2009 Sep 2;16(1):80. doi: 10.1186/1423-0127-16-80 (PMC2741444; doi:10.1186/1423-0127-16-80)

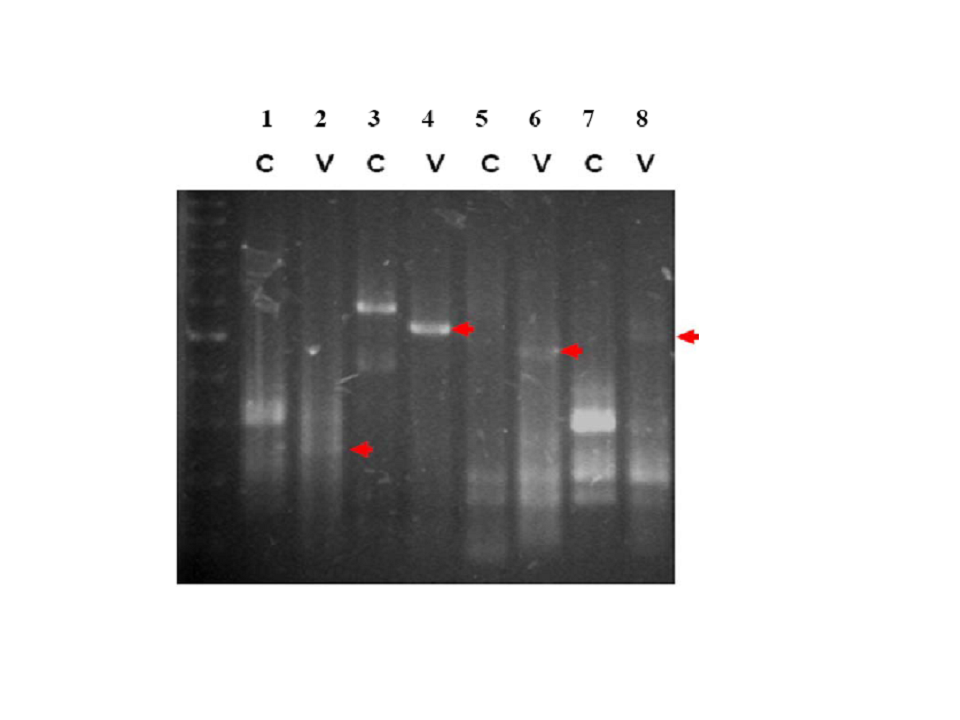

Supplement: Additional file 1 — Supplementary Fig. S1 - Gel analysis of ddRT-PCR products. DNA samples in different lanes represent the outcomes of different sets of primers for PCR (only four sets of primers out of 20 are shown). DNA fragments (marked by arrows) selected to perform T/A cloning and sequencing were cytochrome c oxidase subunt II gene product (lane 2); VV gene products (lanes 4, 6); NADH dehydrogenase subunit IV gene product (lane 8). C: mock-infected HeLa cell; V: 21 hr after vaccinia virus infection (MOI = 1) in HeLa cells. [file 1423-0127-16-80-S1.tiff]

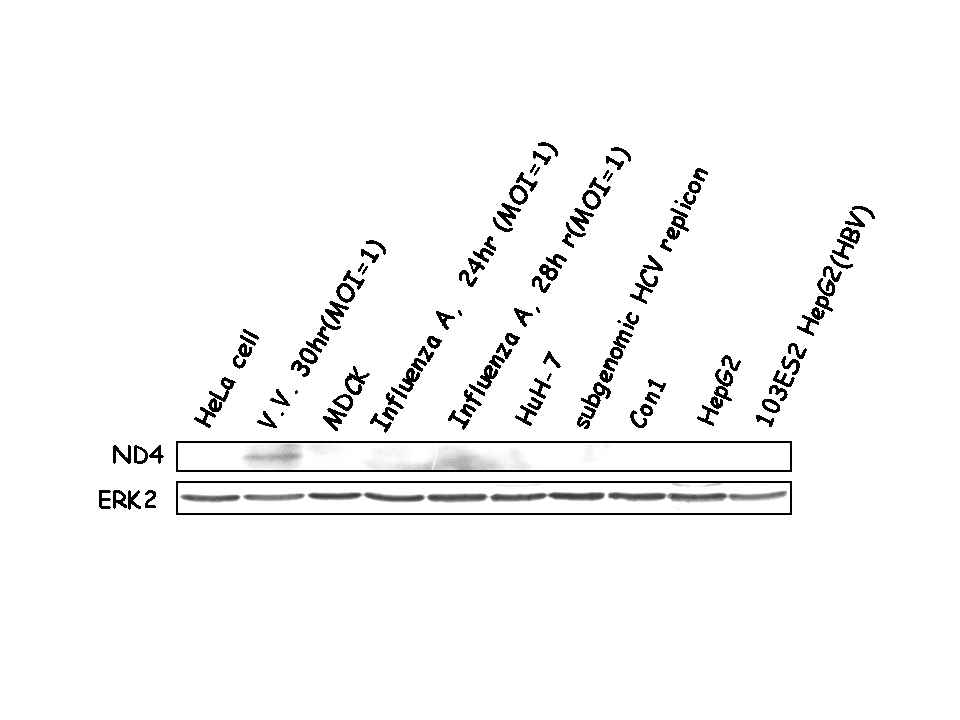

Supplement: Additional file 2 — Supplementary Fig. S2. - Western blotting analysis of ND4 protein expression. Samples were prepared from VV-infected HeLa cells, influenza A virus-infected MDCK cells, HCV replicon cells (with sub-genomic HCV RNA), con1 cells (with full-length HCV RNA) and 1.3 × ES2 cells (with HBV genome). Mock-infected (MDCK cells) or non-transfected parental cells (HuH7 and HepG2 cells) were used as controls. ERK2 protein was used as a loading control. [file 1423-0127-16-80-S2.tiff]

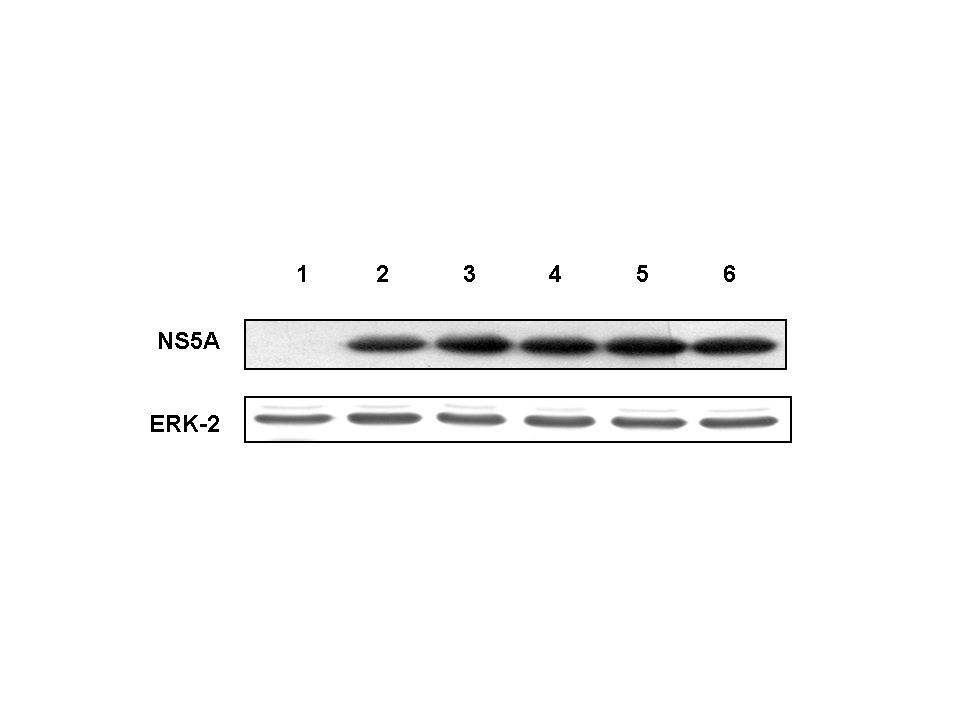

Supplement: Additional file 3 — Supplementary Fig. 3. - Western blotting analysis of NS5A to reflect the amount of HCV sub-genomic RNA. Lane 1, parental HuH7 cell; lane 2, HCV sub-genomic replicon without oligomycin; lane 3, HCV sub-genomic replicon with 3 μM oligomycin; lane 4, HCV sub-genomic replicon with 6 μM oligomycin; lane 5, HCV sub-genomic replicon with 9 μM oligomycin; lane 6, HCV sub-genomic replicon with 12 μM oligomycin. Western blotting was performed 24 hr after oligomycin treatment. Erk-2 protein was used as a loading control. [file 1423-0127-16-80-S3.tiff]

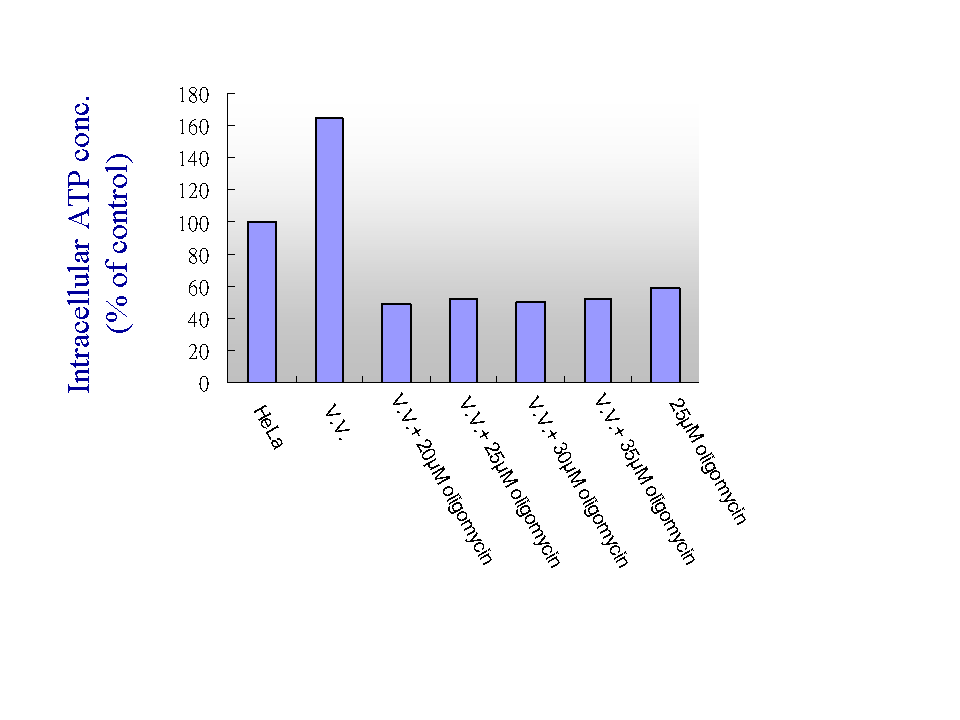

Supplement: Additional file 4 — Supplementary Fig. 4. - Intracellular ATP concentration was measured 13 hr after VV infection (MOI = 1) in the presence of different oligomycin concentrations. Experiments were performed in duplicate. Compared with the ATP level of non-infected and non-treated HeLa cells (as 100%), the ATP level of virus-infected cells was 164% without oligomycin, 49% with 20 μM oligomycin, 52% with 25 μM oligomycin, 49.5% with 30 μM oligomycin, and 52.6% with 35 μM oligomycin. The ATP level was reduced to 58.5% in the presence of 25 μM oligomycin in mock-infected HeLa cells. [file 1423-0127-16-80-S4.tiff]

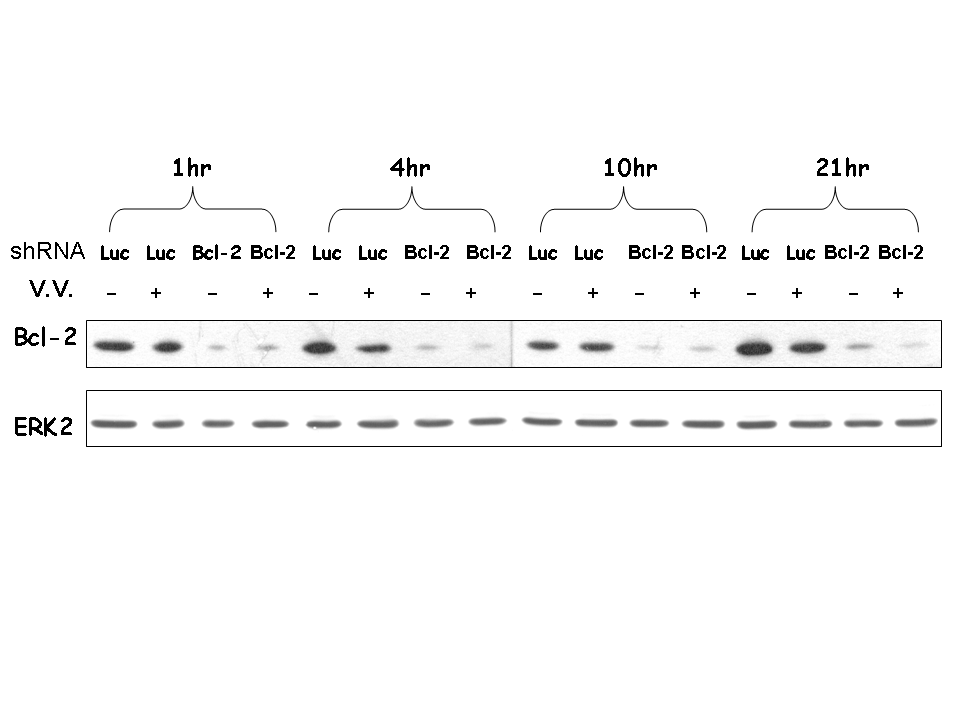

Supplement: Additional file 5 — Supplementary Fig. 5. - Western blotting analysis of Bcl-2 expression in vaccinia virus infected cells. The HeLa cells stably transfected with either shLuc or shBcl-2 (clone 31) were infected with MOI = 1 of vaccinia virus for various time periods, as indicated. ERK2 protein was served as a loading control. [file 1423-0127-16-80-S5.tiff]

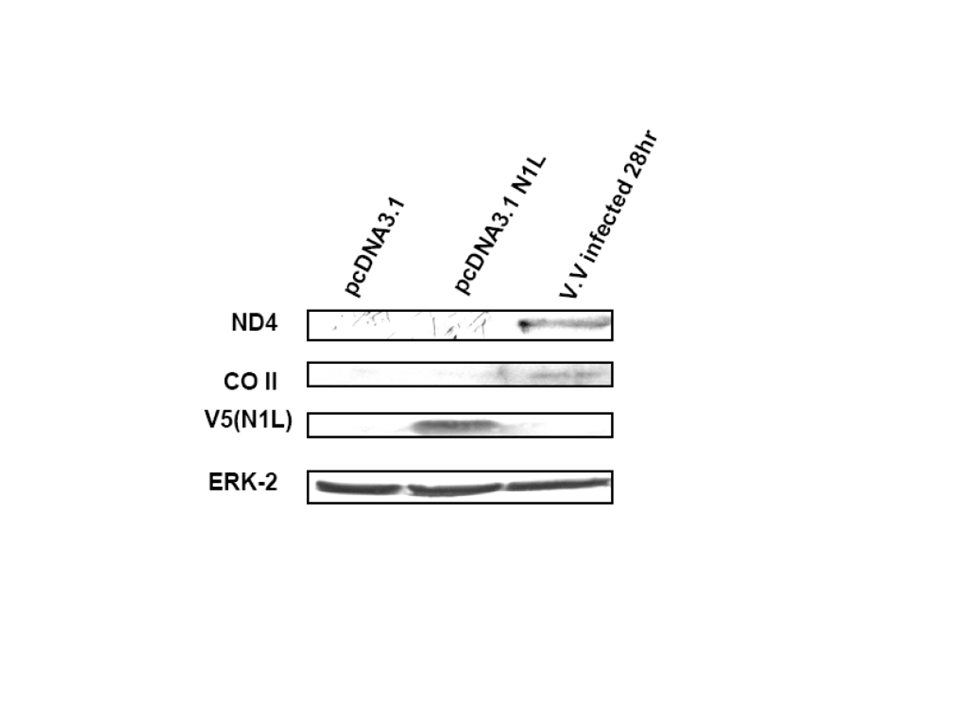

Supplement: Additional file 6 — Supplementary Fig. 6. - Western blotting analysis of ND4 and COII expression in N1L expressed cells. HeLa cells were transfected with expression vector only (pcDNA3.1-V5-HisA) or with N1L expression plasmid (N1L protein with a V5 tag). Cell lysates were analyzed by Western blot 48 hr after transfection. The cell lysate from HeLa cells 28 hr after vaccinia virus infection was used as a positive control, and ERK2 protein was used as a loading control. [file 1423-0127-16-80-S6.tiff]
